# Improvement on mitochondrial energy metabolism of *Codonopsis pilosula (Franch.) Nannf.* polysaccharide

**DOI:** 10.3389/fphar.2025.1545356

**Published:** 2025-05-30

**Authors:** He Li, Letian Zhang, Xingtai Li, Haocheng He, Guoan Fu, Yi Zhun Zhu, Wei Hu, Lige Qiu, Liang Gong, Youming Zhang

**Affiliations:** ^1^ School of Pharmacy, Faculty of Medicine and Faculty of Chinese Medicine, State Key Laboratory of Quality Research in Chinese Medicines, Laboratory of Drug Discovery from Natural Resources and Industrialization, Macau University of Science and Technology, Macau, China; ^2^ Shenzhen Key Laboratory of Genome Manipulation and Biosynthesis, Key Laboratory of Quantitative Synthetic Biology, Shenzhen Institute of Synthetic Biology, Shenzhen Institutes of Advanced Technology, Chinese Academy of Sciences, Shenzhen, Guangdong, China; ^3^ College of Life Science, Dalian Minzu University, Dalian, China

**Keywords:** Codonopsis pilosula (Franch.) Nannf. polysaccharide, mitochondria, energy metabolism, mitochondrial respiratory function, anti-hypoxia, adenosine triphosphate

## Abstract

**Ethnopharmacological relevance:**

*Codonopsis pilosula (Franch.) Nannf.* (CP) is one of the most popular Qi-invigorating herbal medicines and has been extensively used to promote health and vitality in China for a long time. *Codonopsis pilosula (Franch.) Nannf.* polysaccharide (CPP) is the principal active components of CP, which is considered as the reason for CP widespread application. However, it has not been revealed that CPP exert a Qi-invigoration effect by protecting mitochondria and/or improving mitochondrial function in the existing traditional Chinese medicine theories.

**Aim of the study:**

We extracted CPP from *C. pilosula* and investigated the effects of CPP on energy metabolism and mitochondrial protection.

**Methods:**

Based on the mice chronic hypoxia model for imitating the energy deficiency state of the human body, which was administered with CPP by oral gavage daily for 10 days, mitochondrial permeability transition (MPT), lipid peroxidation product malondialdehyde (MDA) in brain, mitochondrial respiratory function, the levels of adenosine triphosphate (ATP), adenosine diphosphate (ADP) and adenosine monophosphate (AMP) in liver cells were assayed. Adenylate energy charge (AEC), total adenylate pool (TAP), ATP/ADP, and ATP/AMP ratios were calculated.

**Results:**

CPP can inhibit the formation of MDA in mice brains, decrease oxygen consuming rate and respiratory control ratio (RCR) of liver mitochondria, increase levels of ATP, TAP and AEC in liver cells under chronic hypoxia condition.

**Conclusion:**

CPP can possess and improve mitochondrial energy metabolism and bioenergetic levels.

## Graphical Abstract

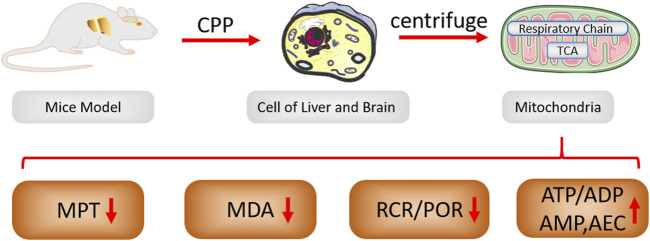

## 1 Introduction

In the field of traditional Chinese medicine (TCM), “Qi” (vital energy) is deemed as the fundamental essence of vitality, embodying the capacity to strengthen the physique, invigorate the spleen, tonify the lung, nourish the blood and engender liquid (Li et al., 2009; Li, 2012), which plays a central role in understanding energy-dependent body functions, and refers to the vital energies or “minute substances” that circulate within the body, encompassing both their physical presence and their functional significance. Viewing from another perspective, it can be considered an expression of the operational state of various organs, which in turn is regulated by complex neuroendocrine mechanisms and energy transformation processes (Ko and Chiu, 2006). Modern medicine believes that the biochemical unit of “Qi” is adenosine 5′-triphosphate (ATP), the mitochondrion becomes the intrinsic source of energy within the cell serving as the cellular powerhouse for ATP synthesis. Mitochondria are the driving force behind life, as mitochondrial oxidative phosphorylation (OXPHOS) provides the main source of energy in the cell. Mitochondria play an essential role in governing both the vitality and death of eukaryotic cells (Cadassou and Jordheim, 2023; Strzyz, 2020). Accordingly, mitochondria represent potential and susceptible hotspots for damage, as the mitochondrion plays a central role in many of the metabolic processes or pathways altered in tumorigenesis (Cadassou and Jordheim, 2023; Schirrmacher, 2020). Unfortunately, the mitochondria are prone to damage due to the reactive oxygen species (ROS) generation during energy transforming process or external stimuli under normal physiological conditions or stressful conditions, when ROS generation exceeds the capacity of antioxidant defenses, oxidative stress ensues and has been implicated in cellular degradation during aging as well as in a variety of disease states (Ko and Chiu, 2006; Lu et al., 2024). The mitochondrial protection is therefore of crucial importance.

According to TCM, the “Qi-invigorating” herbs mainly include *Panax ginseng* (PG), *Codonopsis pilosula (Franch.) Nannf.* (CP), *Astragali radix*, and *Schisandra chinensis*, etc. (Ko and Chiu, 2006; Kwan et al., 2019; Li et al., 2021; Liu et al., 2023; Huang et al., 2018). Among these, PG, a commonly optimal “Qi-invigorating” herb, can combat oxidative stress, affect energy metabolism, and enhance mitochondrial function (Yang et al., 2008; Lan et al., 2022; Ji et al., 2025). However, PG commonly remains expensive as it requires a high growth environment, long growth cycle, and its harvest is difficult, which limits the widespread use of PG. Alternatively, CP, as a substitute for the more expensive PG, has received widespread praise in China, because it possesses some pharmacological activities that PG also provides. CP belonging to the Campanulaceae family, commonly known as “Dangshen” in China, is a perennial species of flowering plant native to Northeast Asia, which is a traditional Chinese tonic medicine with uses for thousands of years (Guo et al., 2024). CP contains a diverse range of pharmacologically active natural compounds, such as polysaccharides, saponins, alkaloids, flavonoids, volatile oil, lignans, terpenoids, etc., and polysaccharides are the main components (Liu et al., 2023; Guo et al., 2024; Sun et al., 2019; Ma et al., 2024). Traditional quality control and evaluation of CP are performed by measuring its polysaccharides content. CPP has anti-tumor, anti-stress, anti-oxidation, enhanced immune function and other activities, making it a key ingredient in CP’s holistic health-promoting effects (Liu et al., 2023; Ma et al., 2024; Chu et al., 2024; Gong et al., 2022; Chen et al., 2024). However, very few reports have systematically measured cell mitochondrial bioenergetics after CPP treatment.

Our previous research mainly focused on the findings of Professor Li Xingtai, which showed that *Panax ginseng* polysaccharide (PGP) was capable of mitigating mitochondrial injury and swelling, consequently enhancing ATP levels and the AEC in liver cells under chronic hypoxia conditions (Li et al., 2009). Other subsequent studies have corroborated these findings, PGP was also found to be related to its ability to promote neuronal mitophagic activity, and the structural degeneration of mitochondria were all ameliorated (Zhang et al., 2023; Wang et al., 2021). These results indicate that PGP protects mitochondria by inhibiting mitochondrial swelling and improving energy status (Li et al., 2009; Wang et al., 2014). The efficacy of CP and PG is so similar that they both contain large amounts of polysaccharide, which is one of their main and key active components, therefore, we guessed that CPP has an analogous effect to those of PGP. To this end, we investigated the protective effects of CPP on mitochondria and ascertained regulation of energy metabolism, further revealed the influence of Qi-invigoration on mitochondrial function, and delineated its underlying mechanism of action, laying the foundation for exploring the essence of “Qi” in the context of traditional Chinese medicine.

## 2 Materials and methods

### 2.1 Animals and materials

All Male mice, weighing 22 ± 2.0 g each, provided with unlimited rodent laboratory chow and drinking water during the experiment period, were purchased from Shenzhen Top Biotechnology Co., LTD. ATP, ADP, AMP, TBA, 1,1,3,3-tetraethoxypropane, DL-malate, L-glutamic acid were purchased from Sigma Chemical (St Louis, MO, United States). while Coomassie Brilliant Blue G-250 (CBBG-250) was sourced from Fluka (Bushs SG, Switzerland). Tris(hydroxymethyl)aminomethane(Tris) was acquired from Gibco BRL (Grand Island, NY, United States). N-2-Hydroxyethylpiperazine-N′-2-ethane sulfonic acid (HEPES) was provided by Merck (Darmstadt, Germany). Bovine serum albumin (BSA) was procured from Boehringer Mannheim Corp. (Indianapolis, IN, United States). All other chemicals and solvents utilized in this study were of analytical grade and manufactured in China. The plant materials of the roots of *C. pilosula* were harvested from the region of Large Xing’an Mountains, which is located in the most northern border of China, Heilongjiang Province, and were identified according to the identification standard of Pharmacopeia of the People’s Republic of China. The plant materials were thoroughly air-dried and finely powdered.

### 2.2 Preparation of CPP

CPP was isolated by hot-water extraction and ethanol precipitation according to the method of Yang et al. (2008) with slight modifications. The dried materials of CP were defatted with 95% alcohol, followed by triple extractions with distilled water at a ratio of 1:10 (g/mL) for a duration of 1 h per extraction in a boiling water bath. After the mixture had been filtered through gauze to collect the filtrate, it was thoroughly mixed and then subjected to evaporation under reduced pressure to concentrate it to a density of 1 g of drug per milliliter. Subsequently, the solution was centrifuged at 3000 revolutions per minute (rpm) for 10 min to sediment any particulates and separate the supernatant, which was carefully collected, and a threefold volume of 95% alcohol was gradually added slowly while stirring continuously to precipitate the polysaccharide, and then refrigerated at 4°C for 24 h, the polysaccharide pellets were obtained by centrifuging at 5000 rpm for 10 min. The polysaccharide pellets were completely dissolved in an appropriate volume of distilled water, deproteinated with Sevag reagent (CHCl_3_:n-BuOH = 4:1, v/v) for 30 min under the magnetic force stirring and the procedure was repeated 3 times, and then centrifuged to remove insoluble material. Finally, the supernatant was lyophilized in the freeze-dry apparatus to give CPP with a brown fluffy shape. The polysaccharides content (92.3%) in extracts was determined using the phenol-sulfuric acid method.

### 2.3 Chronic hypoxia model

Model group and CPP group mice were exposed to hypoxia (10.5% O_2_, 89.5% N_2_) for 10 days in specially constructed plastic cages. The cages were sealed at the top by plastic covers. Small openings were made in the top covers to allow the inflow and outflow of gases and to accommodate water bottles. The oxygen content in the chambers was monitored using a Clark O_2_ electrode inserted through an opening in the top cover. Total gas flow was set at about 1.5 L/min to maintain 10.5% O_2_ in the cage and prevent excessive accumulation of moisture and ammonia. Soda lime was put into the chambers to absorb the CO_2_ which was breathed out by mice. The cages were daily accessed to refresh the bedding and replenish food supplies. Mice in the CPP group received oral gavage at dose of CPP (200, 300 mg/kg/day), in contrast, mice in the model group were administered an equivalent volume of normal saline solution, mice in the normal group were housed in standard open cages (21% O_2_) and given normal saline to serve as a control.

### 2.4 Isolation of liver mitochondria

Mitochondria were obtained using methods described in the literature referred to Fink et al. (2005). Mice livers were swiftly excised immediately and immersed in precooled normal saline to cleanse the surface of residual blood, then placed in an ice-cold isolation medium composed of 0.25 M sucrose, 0.5 mM EDTA and 3 mM HEPES, adjusted to pH 7.4, and homogenized with a motorized Teflon pestle on wet ice. Post homogenization, the samples underwent centrifugation at 1,000 × g for 10 min at 4°C. The supernatants were carefully removed and further centrifuged at 10,000 × g for 10 min. The resulting pellets were then washed twice with the isolation medium and recentrifuged at 10,000 × g for 10 min per wash. After the final wash, mitochondria were resuspended in the isolation medium and kept on ice until further use. Protein concentrations were determined using the Bradford method assay, with BSA serving as the standard reference.

### 2.5 Evaluation of MPT

Liver mitochondria were isolated and resuspended (0.25 mg of protein/mL) in an incubation medium (250 mM sucrose, 1 mM P_i_-Tris, 10 mM Tris-MOPS, 5 mM glutamate-Tris, 2.5 mM malate-Tris, pH 7.4, 25°C). 150 μM Ca^2+^ was added followed by CPP or ruthenium red (0.3 or 0.5 µM) (the model group was excluded). Experiments were started by the addition of 0.5 mg of mitochondrial protein. The final volume was 2 mL. MPT was monitored as the absorbance (A) decrease of the mitochondrial suspension at 540 nm at 0, 2, 5, 10, 15, and 30 min (Walter et al., 2000; He et al., 2000).

### 2.6 Mouse brain homogenate lipid peroxidation assay

Mice were humanely euthanized by cervical dislocation, after which their brains were rapidly extracted, weighed and prepared into 10% (w/v) homogenates with ice-cold normal saline at 0°C to preserve the integrity of the tissue and cellular components. Lipid peroxidation was monitored in terms of MDA using thiobarbituric acid colorimetry (Ohkawa et al., 1979). Briefly, to 0.4 mL homogenate was added 1.5 mL 20% [v/v] acetic acid buffer (pH 3.5), 0.2 mL of 8.1% [w/v] sodium dodecyl sulphate, 1 mL of 0.67% TBA (w/v) and 0.4 mL water, the tubes were incubated at 95°C for an hour, cooled with running tap water, and were extracted with 5 mL n-butanol. After centrifugation (2,000 × g, 10 min), the absorbance of the butanol phase was read at 532 nm. MDA were determined by linear regression analysis of a standard aliquot using 1,1,3,3-tetraethoxypropane as a standard.

### 2.7 Measurement of liver mitochondrial respiratory function

Liver mitochondrial respiratory function was measured utilizing the method described by Estabrook. Oxygen consumption was measured at 30°C within a sealed, stirred, and thermostatted glass vessel, fitted with a Clark-type oxygen electrode, in a 2.0 mL respiration medium buffer (pH 7.4), which was composed of 225 mM sucrose, 1 mM EDTA, 5 mM MgCl_2_, 15 mM KCl, 15 mM KH_2_PO_4_, 50 mM Tris,5 mM L-glutamic acid, 10 mM DL-malate, and 5 mg/mL mitochondrial protein. Respiratory state 3 (S3) was defined as the oxygen (O_2_) consumption rate of mitochondria in the presence of substrate after the addition of 0.25 mM ADP, a potent stimulator of mitochondrial respiration. Respiratory state 4 (S4) was characterized as the oxygen (O_2_) consumption rate when all the ADP has been phosphorylated. The rates of S3 and S4 can be precisely determined by analyzing the OXPHOS curve. Respiration rates as nanomoles of oxygen atom consumed per minute per milligram of protein. The RCR was calculated as the ratio of S3 to S4 respiration rates, which provided insight into the coupling efficiency of OXPHOS. The P/O (the number of moles of Pi consumed for each oxygen atom reduced to H_2_O) ratio is equivalent to the number of ADP molecules phosphorylated per oxygen atom reduced, reflecting the stoichiometry of ATP synthesis in relation to oxygen reduction.

### 2.8 Measurement of ATP, ADP, and AMP in liver cells by HPLC

The measurement of ATP, ADP, and AMP in liver cells was conducted based on our previously established methodology (Li et al., 2009), by gradient RP-HPLC (Spherisorb C18 reversed-phase chromatographic column, 4.6 mm × 250 mm, 5 µm particle size)with an ultraviolet detector at ambient temperature, and with mobile phase flow rate of 0.8 mL/min. The gradient system utilized two mobile phases: buffer A, a 0.05 M KH2PO4-K2HPO4 solution adjusted to pH 6.0, and buffer B, which was buffer A supplemented with 10% methanol (v/v). The gradient elution procedure was as follows: from 0 to 3 min, buffer A served as the mobile phase; between 3 and 6 min buffer A was gradually reduced from 100% to 0% while buffer B increased from 0% to 100%; between 6 and 9 min, buffer B was the sole mobile phase; and after 9 min, buffer A was reintroduced as the sole mobile phase, with the total run time being 12 min, when the detection wavelength was set at 254 nm. The contents of ATP, ADP, and AMP within liver cells were determined by computing the peak areas obtained from chromatographic analysis with those of standard nucleotide solutions of established concentrations. The TAP and AEC were subsequently calculated to provide a comprehensive assessment of the cellular energy state, using the following formulas respectively:

$$\text{TAP}=\left[\text{ATP}\right]+\left[\text{ADP}\right]+\left[\text{AMP}\right]$$

$$\text{AEC}=\left(\left[\text{ATP}\right]+0.5\left[\text{ADP}\right]\right)/\text{TAP}$$

### 2.9 Statistical analysis

Data were presented as means ± SEM, and statistical differences among groups were evaluated using one-way analysis of variance (ANOVA), complemented by the least significant difference (LSD) *post hoc* test for multiple comparisons. These analyses were conducted using the SPSS 16.0 statistical software package for Windows (SPSS Inc., Chicago, Illinois, United States). The results were considered statistically significant at a probability (P) value threshold of less than 0.05.

## 3 Results

### 3.1 CPP inhibited Ca^2+^-induced liver MPT *in vitro*

**FIGURE 1:**
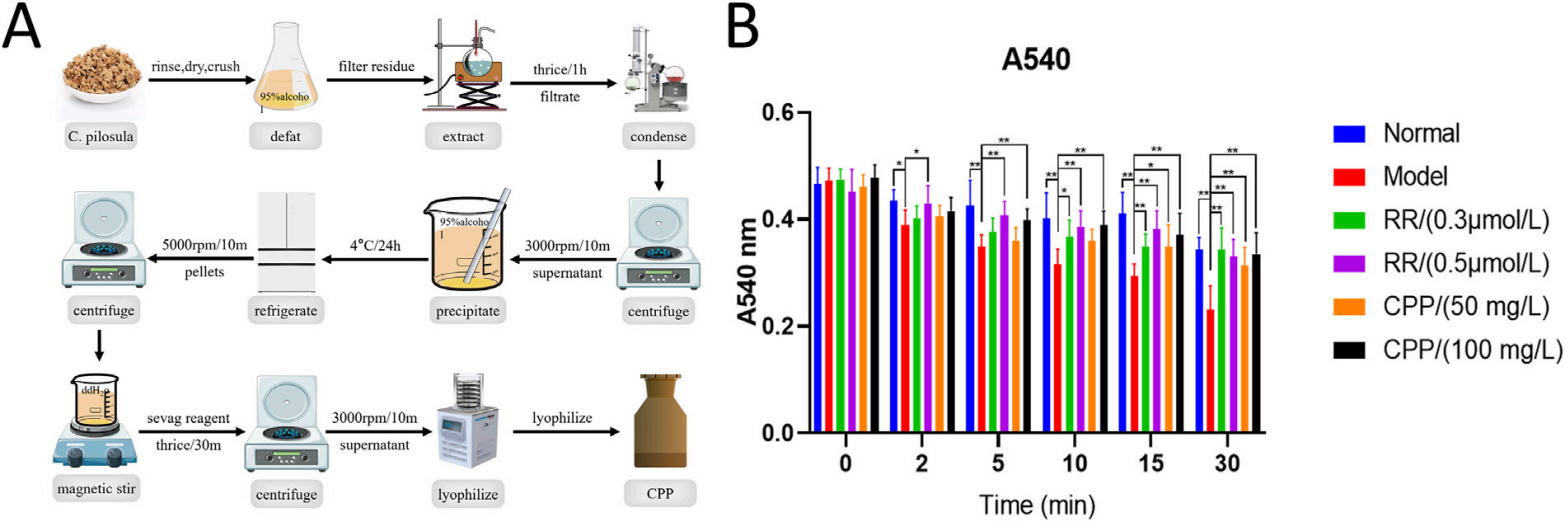
**(A)** Preparation of CPP. **(B)** CPP’s inhibition effects (50 mg/L, 100 mg/L) on Ca^2+^-induced liver MPT *in vitro*. A_540 nm_: Absorbance at 540 nm; RR: ruthenium red, CPP: *Codonopsis pilosula (Franch.) Nannf.* polysaccharide. MPT: mitochondrial permeability transition.; All values are mean ± SEM (n = 10). ^*^ *P* < 0.05, ^**^ *P* < 0.01, compared with model group.

As is well known, mitochondrial energy metabolism is a complex system in where biochemical reactions are coupled to membrane electrophysiology, the normal function of mitochondria is highly dependent on their fluidity and integrity. The opening of the mitochondrial permeability transition pore (MPTP) significantly reduces mitochondrial membrane potential (MMP/Δψm), further damaging mitochondrial function (Li et al., 2009; Li, 2012) Mitochondrial calcium overload can trigger the opening of the MPTP, causing uncoupling of OXPHOS, swelling of the mitochondria due to water influx, and rupture of the mitochondrial outer membrane (Carraro and Bernardi, 2023; Bauer and Murphy, 2020). Here, we extracted CPP from CP using combined hot-water extraction with ethanol precipitation (Figure 1A), and then isolated the mice liver mitochondria by differential centrifugation and constructed the Ca^2+^-induced MPTP openness model. The MPTP can be monitored via mitochondrial permeabilization to sucrose based on the changes of absorbance at 540 nm (Carraro and Bernardi, 2023; Bauer and Murphy, 2020). The harvested mitochondria treated with CPP or Ca^2+^ blocker ruthenium red (RR) were challenged with a Ca^2+^ load of 150 µM. In the model group, Ca^2+^ can decrease significantly the absorbance of 540 nm on 2 min, which caused a detectable MPTP, indicating the rapid and large amplitude mitochondrial swelling induced by Ca^2+^ (Figure 1B). Interestingly, the effects of Ca^2+^ on MPTP were completely blocked by 0.5 µM RR and partially blocked by 0.3 µM RR (Figure 1B). Fortunately, CPP also obviously inhibited Ca^2+^-induced MPTP openness, and the inhibitory potency was stronger when the incubation time was longer and the concentration of CPP was higher (Figure 1B). No significant difference was observed between the CPP group (100 mg/L) and the normal group (Figure 1B), suggesting CPP can completely resist the toxicity of Ca^2+^ overload on mitochondria, allowing mitochondria to maintain normal function.

### 3.2 CPP decreased MDA formation under chronic hypoxia *in vivo*

**FIGURE 2:**
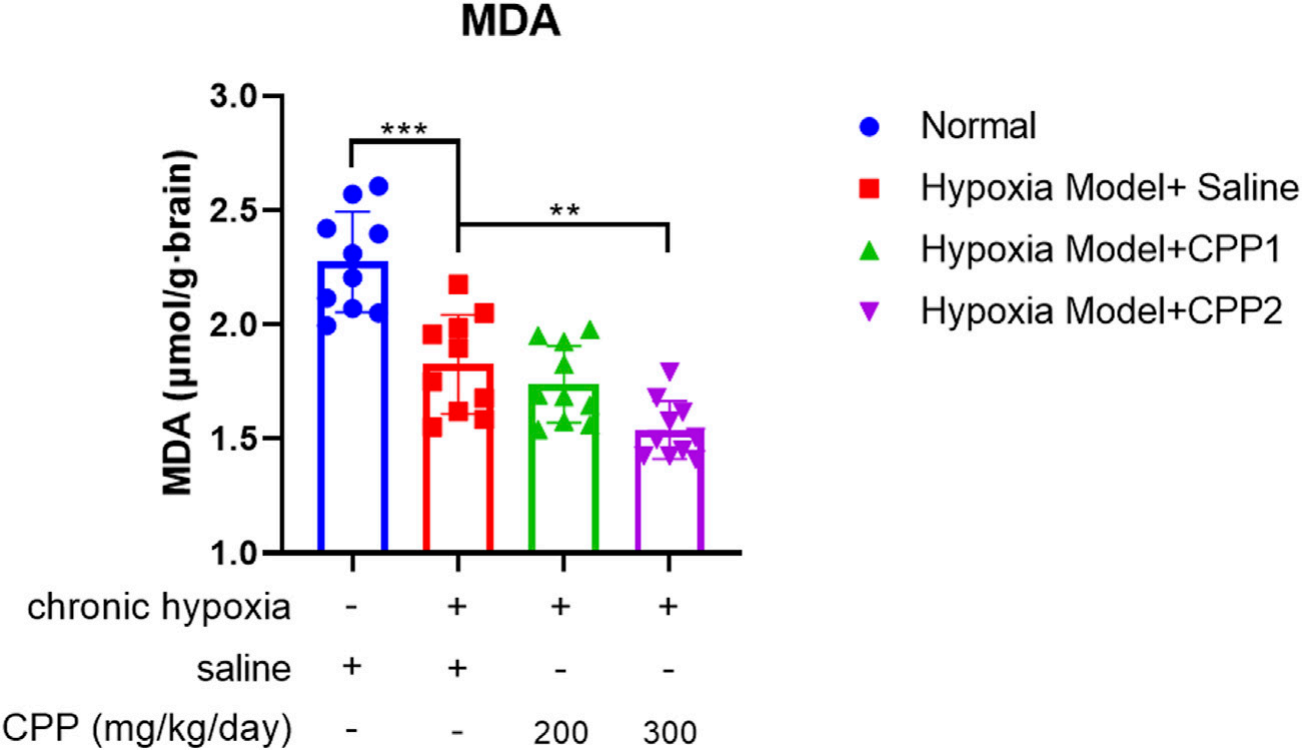
The effects of CPP on brain MDA level *in vivo*. CPP significantly decreased MDA formation in brain mitochondria. All values are mean ± SEM (n = 10). **P < 0.05, ***P < 0.001 compared to model group.

Most ROS formation occurs at the intracellular oxygen-consumed site, which means that the predominant site of ROS generation is mitochondria (Fuhrmann and Brüne, 2017). When mitochondria undergo OXPHOS, the aberrant O_2_ reactions during electron transport induce ROS byproduct generation. In addition, the production rate of ROS is positively correlated with the concentration of environmental O_2_. The ROS overproduction can cause peroxidation of the polyunsaturated fatty acids (PUFA) in the mitochondrial membrane, leading to the formation of lipid peroxides such as MDA (Valgimigli, 2023; Hajieva et al., 2023). The MDA, owing to its high cytotoxicity and inhibitory action on protective enzymes, is a biomarker of the cellular ferroptosis (Cui et al., 2022). Therefore, the MDA level is an important marker of mitochondrial function. We established the saline or CPP-treated mice chronic hypoxia model (10.5% O_2_) and the saline-treated normal O_2_ (21% O_2_) mice group model, and then analyzed the MDA level of these mice brain mitochondria. The MDA level in the chronic hypoxia model group was significantly lower than that in the normal O_2_ group, as the decrease in O_2_ significantly reduced ROS generation, further diminishing MDA production. Notably, CPP administration extremely inhibited MDA formation in brain mitochondria and exhibited concentration dependence under the chronic hypoxic condition (Figure 2), indicating the antioxidant activity of CPP in mitochondria.

### 3.3 The effects of CPP on liver mitochondrial respiratory function *in vivo*

**FIGURE 3:**
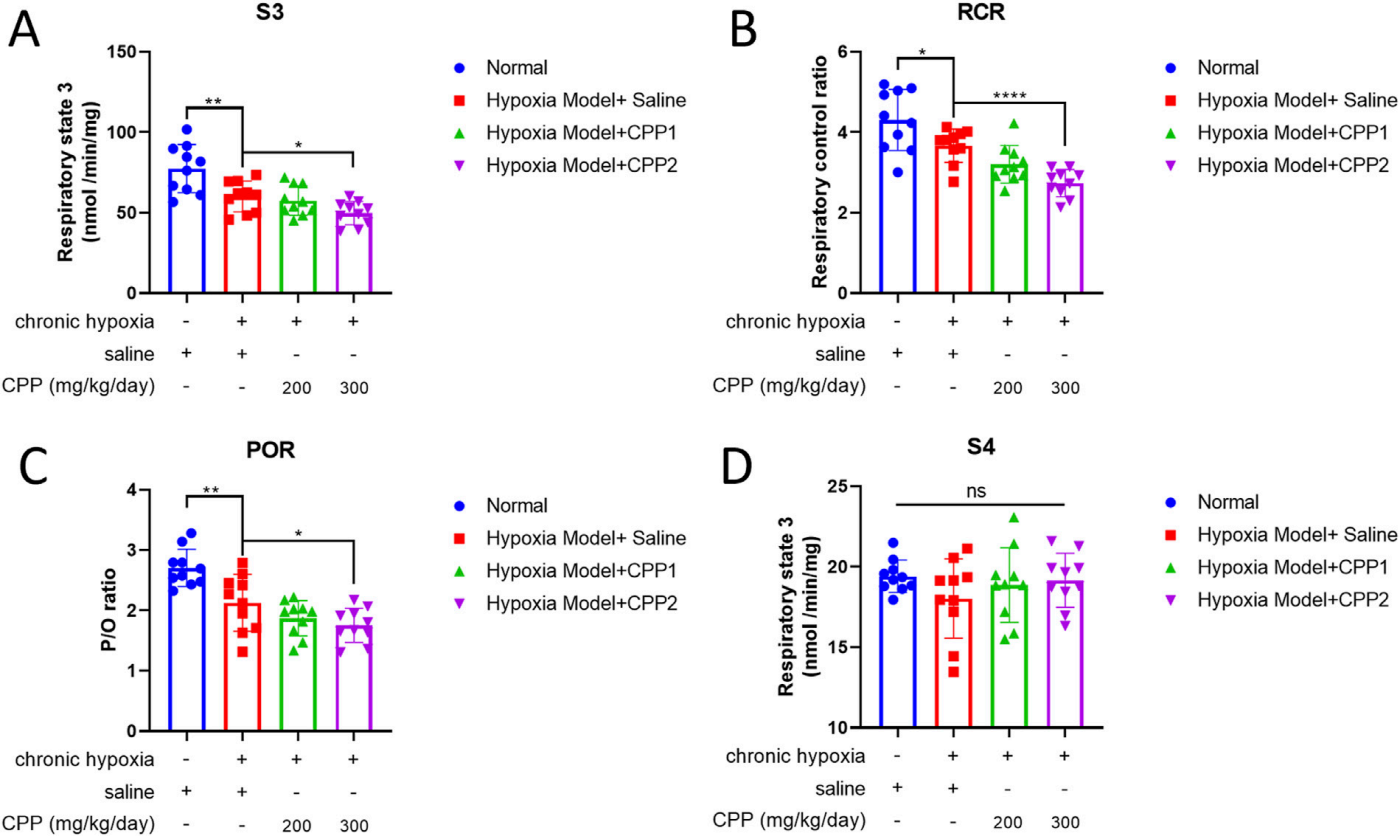
The effects of CPP on liver mitochondrial respiratory function *in vivo*. **(A)** S3: Respiratory state 3. **(B)** RCR: Respiratory control ratio. **(C)** P/O: The number of moles of Pi consumed for each oxygen atom reduced to H_2_O. **(D)** S4: Respiratory state 4. All values are mean ± SEM (n = 10). *P < 0.05, **P < 0.01, ****P < 0.0001 compared to model group.

The liver is very instrumental in metabolic processes, pivotal for sustaining energy levels and ensuring structural stability of the body. The mitochondria isolated from hepatocytes are widely used in many biochemical studies. To assay the CPP protection of mitochondrial respiratory function, we detected S3 (O_2_ consumption rate after adding ADP) and the S4 (O_2_ consumption rate after conversion ADP to ATP via OXPHOS reaction) of liver mitochondria from chronic hypoxia mice model and then calculated the RCR values and P/O ratio (POR). Compared to normal group mice, hypoxic mice (model group) showed a significant decrease in S3, RCR and POR (Figures 3A–C), suggesting a decrease in oxygen consumption rate and a robust reduction in the rate of ATP generation through ADP phosphorylation during the exposure in chronic hypoxia. CPP could further reduce these parameters (Figures 3A–C), whereas there was no significant effect on S4 (P > 0.05) (Figure 3D).

### 3.4 The effects of CPP on energy state of mice hepatocyte under chronic hypoxia *in vivo*

**FIGURE 4:**
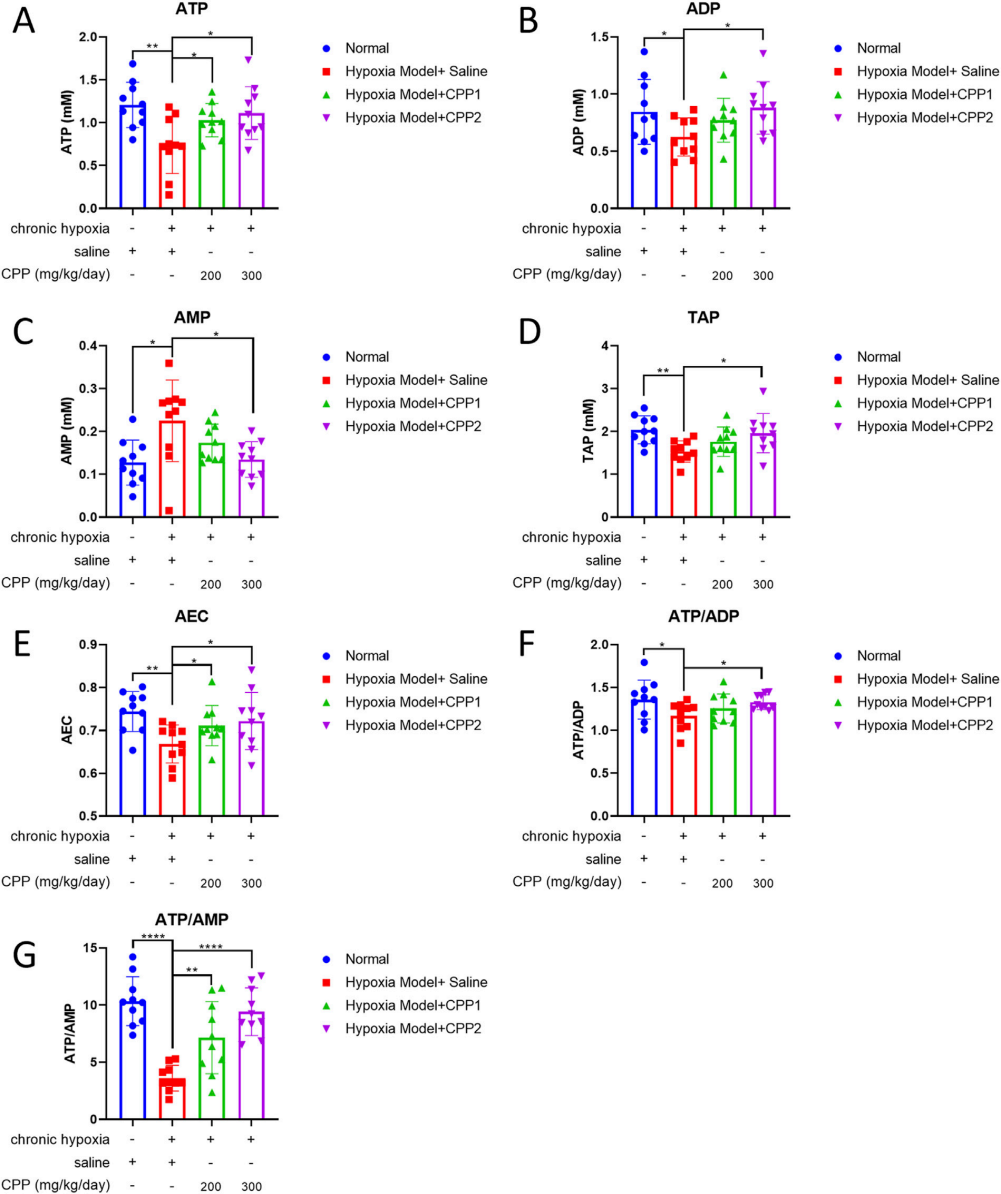
Effects of CPP on energy status of mice hepatocyte *in vivo*. **(A)** ATP level. **(B)** ADP level. **(C)** AMP level. **(D)** TAP level. **(E)** AEC level. **(F)** ATP/AEP. **(G)** ATP/AMP. All values are mean ± SEM (n = 10). *P < 0.05, **P < 0.01 compared to model group.

ATP is a direct supplier of energy required within cells. Insufficient supply of ATP can have serious adverse consequences on energy dependent metabolic pathways, and energy depletion can lead to cell death. The abovementioned studies showed that CPP can reduce the O_2_ consumption rate of mitochondria under hypoxic conditions, but it is unclear whether this process will affect the generation of mitochondrial ATP. Our research identified that hypoxia led to a marked fall in cellular ATP and ADP levels (Figures 4A,B), and a rise in cellular AMP levels (Figure 4C) associated with reductions in ATP/ADP and ATP/AMP ratios, alterations in ATP/ADP ratio could substantially impact the MMP (∆Ψ_m_). The cellular AMP/ATP ratio serves as a biomarker, indicative of metabolic stress. Through the catalytic action of adenylate kinase (AK), any reduction in the cellular ATP/ADP ratio is mirrored by a corresponding decrease in the ATP/AMP ratio. Hypoxia induces a significant decline in the ATP/AMP ratio, which was observed to drop from 10.46 under normoxic conditions to 3.22 under hypoxic conditions, while the ATP/ADP ratio decreased from 1.39 to 1.18 (Figures 4F,G). These findings indicate that the hypoxia significantly altered the cellular energy state.

The AEC serves as a quantitative indicator that delineates the proportion of ATP and its precursors within the adenine nucleotide system, offering a linear measure of the metabolic energy status within cells. TAP is another gauge of cellular energy status, which was observed to be decreased in liver cells of the model group compared with the normal group in our study (Figure 4D). Furthermore, the AMP level in the model group was observed to be twice as high as that in the normal group (Figure 4C). Treatment with CPP (300 mg/kg/day) could increase ATP, ADP, TAP levels and ATP/ADP, ATP/AMP ratio, AEC of liver cells compared to the model group (Figures 4A,B,D–G). The data showed CPP to be an enhancer of ATP production under hypoxia-induced anti-ATP circumstance, while ATP levels were drastically lowered by hypoxia but CPP stimulated an increased output of ATP. Notably, ATP/AMP ratio in the CPP (300 mg/kg/day) group is increased over 2-fold than the model group (Figure 4G), indicating that CPP-treated mice mitochondria exhibited higher production capacity efficiency under chronic hypoxia, despite the lower O_2_ consumption rate.

## 4 Discussion

In the hub of cellular bioenergetics, mitochondria are key players in regulating cellular energy, which underlies the metabolic and functional changes of cells (Infantino et al., 2021). Mitochondria are important and pivotal regulators of cell death, responding to a wide variety of stress signals, including loss of growth factors, hypoxia, oxidative stress, and DNA damage. Mitochondria are also considered the pacemakers of tissue aging due to the continuous production of free radicals, oxygen, and nitrogen free radicals and related reactive species, and to the selective oxidative damage that leads to mitochondrial dysfunction (Fuhrmann and Brüne, 2017). Modern TCM science has established a certain connection between mitochondrial protection and nourishing “Qi,” which is the most vital force for retaining the physiological functions of the human body (Li et al., 2009; Li, 2012; Huang et al., 2019; Valcarcel-Jimenez et al., 2017). For instance, the PGP improved the tolerance of mitochondria to oxidative damage through the inflammatory response, oxidative damage and signaling pathway, just as increasing TAP, MMP and antioxidant capacity reflected by superoxide dismutase (SOD), glutathione (GSH) and so on (Li et al., 2021; Wang et al., 2021, Huang et al., 2019). CP is also known for its ability as a substitute of costly PG, given PG’s well-established antioxidant and mitochondrial protective properties. Among them, CPP is the primary active components of CP, the quality of CPP was evaluated by HPGPC profiling and chemometrics, which showed that CPP could be further purified and two different molecular weight polysaccharides (CP-1 and CP-2) were obtained, which contained 80.32% and 79.05% of total sugars, with the average molecular weights of 2568.51 kDa and 3.20 kDa (Liu et al., 2025). CPP is a typically acidic heteropolysaccharide, including arabinose, glucose, rhamnose, galactose, mannose, glucuronic acid and galacturonic acid in the mole percentages of 13.9, 29.8, 4.6, 14.0, 2.2, 1.2% and 34.3% (mol%) respectively (Yang et al., 2008; Yang et al., 2024). Although recent studies demonstrated that CPP protected the mitochondrial membrane integrity of the sheep sperm after preservation at 4°C (Wang et al., 2024), CPP may inhibit hepatocellular carcinoma growth and reestablish the immune balance (Chen et al., 2024; Tang et al., 2021; Li et al., 2024), and CPP increased NAD+, NAD+/NADH, and PGC-1α related to NAD+, thus partially recovering ATP (Li et al., 2024; Hu et al., 2021), it is still unclear whether CPP can also achieve the “Qi-invigoration” through mitochondrial protection in TCM theory. The integrity of mitochondria is a prerequisite for the energy production through OXPHOS. Furthermore, mitochondrial calcium overload might trigger the opening of the MPTP, causing uncoupling of OXPHOS, swelling of the mitochondria due to water influx, and rupture of the mitochondrial outer membrane (Bauer and Murphy, 2020). Our study demonstrated that CPP can inhibit MPTP openness during Ca^2+^-challenge. CPP can scavenge superoxide anion and hydroxyl radicals, enhancing the SOD activity (Ma et al., 2024; Chen et al., 2024; Feng and Zhang, 2020). The inhibition of CPP on MPTP might be closely related to its scavenging activity on ROS and the inhibition on lipid peroxidation, this indicates that CPP may protect mitochondria by scavenging ROS and antioxidation properties.

Oxidizing agents and ROS lead to the opening of MPTP, whose immediate consequence is the collapse of ∆Ψm, besides increasing matrix volume, leading to major modifications of mitochondrial function and structure that eventually jeopardize the maintenance of cell viability. And lipid peroxides (such as MDA) can increase membrane permeability, leading to mitochondrial swelling. The present results showed that CPP was a potent inhibitor of MDA, which could decrease lipid peroxides extent of brain. Previous reports CPP can scavenge superoxide anion and hydroxyl radicals, enhance the SOD activity and decrease MDA content in mouse brain. Mitochondrial oxidative stress has been implicated in cell death (Chen et al., 2024; Orrenius et al., 2007), high levels of pro-oxidants produced by mitochondria can induce apoptosis by changing cellular redox status, depleting reduced GSH, reducing ATP levels, and decreasing reducing equivalents such as NADH and NADPH (Orrenius et al., 2007). Either Qi deficiency or hypoxia can markedly inhibited the activity of SOD and GSH (Li et al., 2020), decrease ATP, TAP and AEC, increase AMP content, and these severe cases cause the excessive decrease of ATP levels, leading to rapid cell necrosis. Our investigation found that CPP (300 mg/kg/day) can further decrease state 3 respiration, RCR, and POR of liver mitochondria compared to the control group in our experimental model. We think that the treatment of CPP reduces the energy consumption of the body during hypoxia, thus playing a role in nourishing “Qi.” When hypoxia occurs, hypoxia-inducible factor 1 (HIF-1) is a pivotal modulator of the metabolic reprogramming (Fuhrmann and Brüne, 2017; Infantino et al., 2021), the utilization rate of oxygen and ATP production efficiency are significantly improved, and the production of oxygen byproduct ROS is significantly inhibited during electron transfer. Although the results of these experiments demonstrate the efficacy of CPP, it should be noted that direct hypoxia markers, such as HIF-1α expression would strengthen model validation, these assays were not included here, this limitation will incorporate analysis in future studies.

Researchers have elucidated a secondary mechanism of respiratory control in eukaryotic cells (Kadenbach et al., 2010), which is regulated by the intramitochondrial ATP/ADP ratio. Under conditions of high ATP/ADP ratio, OXPHOS is inhibited due to the allosteric binding of ATP to a specific subunit of Complex IV, and this inhibition can be counteracted or reversed by increased ADP concentrations, thus modulating the rate of OXPHOS in response to cellular energy demands. Cell energy metabolism is further regulated by AEC. We also discovered that CPP could increase the levels of ATP and TAP in hypoxic liver cells, reduce AMP levels, and consequently increase ATP/ADP, ATP/AMP ratios and AEC (i.e., increase cellular bioenergetics). We consider this is the result from the alleviation of feedback inhibition on OXPHOS, which in turn ameliorates mitochondrial energy metabolism and elevates the bioenergetic level (Kadenbach, 2020). Critical mitochondrial functions, including ATP synthesis, ion homeostasis, metabolites transport, ROS production, and cell death are highly dependent on MMP (Solaini et al., 2007; Kowaltowski and Abdulkader, 2024). The energy state of the mitochondria can retro-regulate the nuclear-encoded energy genes. Variations in the activity of the mitochondrial respiratory chain are accompanied by changes in “energy-state messengers,” which encompass ROS (such as the diffusive H_2_O_2_), mitochondrial and cytosolic calcium, NADH/NAD+, ATP/ADP, GTP, AMP, cyclic AMP (cAMP), Δψm and ΔpH (Benard et al., 2010). In addition, an increase in Δψm, whether caused by impaired OXPHOS or by an overabundance of nutrients relative to ADP, will result in aberrant electron migration in the electron transport chain and elevated ROS production (Wallace, 2005). From both an economic and health perspectives, multicellular organisms necessitate a regulatory mechanism that operates independently of the Mitchell theory (chemiosmotic theory), while maintaining the Δψm at moderately low levels of 120–140 mV (Begum and Shen, 2023), thereby optimizing the efficiency of OXPHOS. Surprisingly, the German scientist Kadenbach et al. (2010) have proposed an innovative mechanism that bypasses the Mitchell theory, in which a high ATP/ADP ratio exerts feedback inhibition on CcO (complex Ⅳ) (Kadenbach, 2020), and maintains a low Δψm value, thereby preventing ROS generation and preserving the high efficiency of OXPHOS. This novel mechanism represents a new extension of Mitchell theory, known as “The second mechanism of respiratory control,” offering a fresh perspective on how cells modulate their respiratory activity.

Mitochondria play a pivotal role in governing the cellular life and death by intricately manipulating and regulating several critical factors, such as bioenergetics, the MPTP, and the mitochondrial redox-status (Guo, 2021; Meng et al., 2025; Purandare et al., 2023; Zorova et al., 2018). Mitochondria consume over 90% of the oxygen utilized in cells and are the major source of cellular ROS production, which are produced in substantial quantities through aberrant O_2_ reactions with the components of the electron transport chain, which is stringently controlled in physiological conditions. And the majority of ROS production remain tightly confined inside intact mitochondria, they appear to be more susceptible to bear the brunt of the free radical damage observed in cells during aging. This susceptibility is evident in the increased oxidative stress observed in aging cells, which is associated with a decline in respiratory function and is a key factor. This phenomenon can be a result of an escalating cycle, whereby impaired mitochondria leakage increased levels of free radicals, thereby causing further self-inflicted damage as well as extending the deleterious effects to the rest of the cell. The rate of mitochondrial respiration and the associated of ROS production are substantially influenced by the coupling state of the mitochondria, which refers to the efficiency with the electron transport chain generates ATP relative to the amount of oxygen consumed. And mitochondrial metabolism can be both advantageous and detrimental to these processes, which keep a balance that the dualities of mitochondria is an adaptive homeostasis mechanism (Valcarcel-Jimenez et al., 2017; Yu and Pekkurnaz, 2018; Wu et al., 2022). In the current study, CPP demonstrated the ability to reduce the oxygen-consuming rate and RCR in liver mitochondria, thereby increasing hypoxia tolerance and prolonging the survival time of mice. This improvement observed in mitochondrial function is attributed to CPP’s capacity to bolster cellular bioenergetics, thereby optimizing the mitochondria’s role in energy metabolism and cellular resilience under oxygen-deprived conditions. We consider that this effect is interpreted as a reduction in the basal metabolic rate, which can be seen as a protective adaptive mechanism. Patients with Qi deficiency, who exhibit increased susceptibility to fatigue and diminished energy levels, require nutritional supplementation, sufficient rest and a reduction in energy expenditure. According to the current study, we propose that CPP can mitigate the symptoms associated with Qi deficiency by enhancing mitochondrial bioenergetics, while higher levels of bioenergy will exert a feedback inhibition on OXPHOS, potentially contributing to the metabolic conservation and cellular protection (Kadenbach, 2020). Consequently, This study significantly expands Kadenbach’s “New extension of the Mitchell Theory” through systematic analysis of multiple bioenergetic parameters. This not only provides compelling evidence supporting the contention that “the second mechanism of respiratory control constitutes a novel extension of the Mitchell Theory in OXPHOS,” but also proposes a potential intervention strategy by CP for cellular bioenergetic enhancement.

However, we have recognized some limitations of this research. as a theoretical basis and novel ideas were provided for Qi-invigoration, using mitochondrial bioenergetics as a target, this study is subject to these inherent limitations. First, while therapeutic efficacy is holistically assessed within the paradigm of TCM, the experimental design employed conventional single-variable control methodologies predominant in contemporary Western scientific paradigms. Second, the absence of MMP measurements using JC-1 or TMRE fluorescent probes to quantify CPP’s effects on MMP stabilization and ROS reduction (Kowaltowski and Abdulkader, 2024; Popgeorgiev et al., 2024), this may fall short of elucidating the intricate synergies. Although this investigation establishes a theoretical foundation for Qi-invigoration strategies targeting mitochondrial bioenergetics, these inherent constraints suggest that complete elucidation of multilevel therapeutic synergies may require further integrative research approaches.

## 5 Conclusion

**FIGURE 5:**
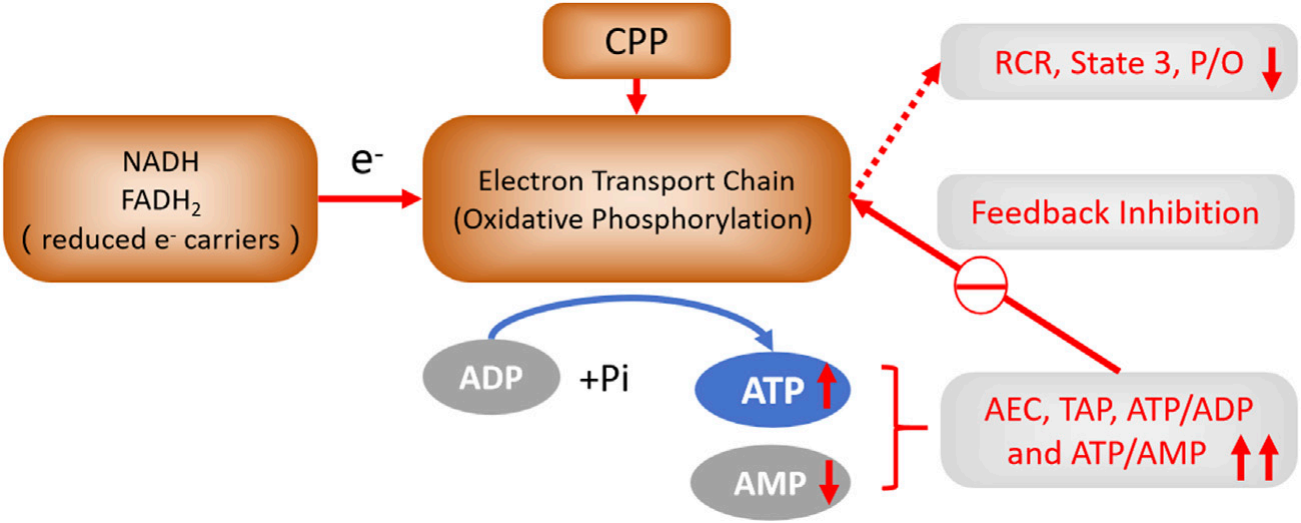
The feedback regulation of CPP on OXPHOS. CPP can decrease AMP, and increase ATP, TAP, ATP/ADP, ATP/AMP ratio, AEC in hypoxic liver cells, which feedback inhibit OXPHOS by decreasing RCR, S3 and POR of liver mitochondria, this may further decrease Δψm to reduce ROS generation. This outcome is attributed to the enhancement of mitochondrial energy metabolism and the elevation of cellular bioenergetic levels.

In summary, we proved that CPP had the pharmacological activities of antihypoxia, antioxidation and mitochondrial protection, and concluded that the enhancement of mitochondrial energy metabolism and bioenergetic levels via CPP administration may be the biological mechanisms to invigorate Qi (Figure 5).

## Data Availability

The raw data supporting the conclusions of this article will be made available by the authors, without undue reservation.

## Abbreviations

CP, *Codonopsis pilosula (Franch.) Nannf.*; CPP, *Codonopsis pilosula (Franch.) Nannf.* polysaccharide; PG, *panax ginseng*; PGP, *panax ginseng* polysaccharide; TCM, raditional Chinese medicine; MMP/Δψm, mitochondrial membrane potential; MPT, mitochondrial permeability transition; MPTP, mitochondrial permeability transition pore; MDA, malondialdehyde; OXPHOS, oxidative phosphorylation; ATP, adenosine triphosphate; ADP, adenosine diphosphate; AMP, adenosine monophosphate; AEC, adenylate energy charge; TAP, total adenylate pool; RCR, respiratory control ratio; P/O, the number of moles of Pi consumed for each oxygen atom reduced to H_2_O; POR, P/O ratio; ROS, reactive oxygen species; SOD, superoxide dismutase; S3, respiratory state 3; S4, respiratory state 4; GSH, glutathione.
