# Correction: Improvement on mitochondrial energy metabolism of *Codonopsis pilosula (Franch.) Nannf.* polysaccharide

**DOI:** 10.3389/fphar.2025.1638311

**Published:** 2025-07-14

**Authors:** He Li, Letian Zhang, Xingtai Li, Haocheng He, Guoan Fu, Yi Zhun Zhu, Wei Hu, Lige Qiu, Liang Gong, Youming Zhang

**Affiliations:** ^1^ School of Pharmacy, Faculty of Medicine and Faculty of Chinese Medicine, State Key Laboratory of Quality Research in Chinese Medicines, Laboratory of Drug Discovery from Natural Resources and Industrialization, Macau University of Science and Technology, Macau, China; ^2^ Shenzhen Key Laboratory of Genome Manipulation and Biosynthesis, Key Laboratory of Quantitative Synthetic Biology, Shenzhen Institute of Synthetic Biology, Shenzhen Institutes of Advanced Technology, Chinese Academy of Sciences, Shenzhen, Guangdong, China; ^3^ College of Life Science, Dalian Minzu University, Dalian, China

**Keywords:** *Codonopsis pilosula (Franch.) Nannf.* polysaccharide, mitochondria, energy metabolism, mitochondrial respiratory function, anti-hypoxia, adenosine triphosphate

The following funders were erroneously omitted from the **Funding** statement: “Natural Science Foundation of China (T2250710184); Shenzhen Science and Technology Program (ZDSYS20220303153551001); the Fundamental Research Funds for the Central Universities in China (C10030105)”.

The correct **Funding** statement appears below.

**Funding**

The author(s) declare that financial support was received for the research and/or publication of this article. This study was supported by the Natural Science Foundation of China (T2250710184); Shenzhen Science and Technology Program (ZDSYS20220303153551001); the Fundamental Research Funds for the Central Universities in China (C10030105); Macau Science and Technology Development fund [FDCT (0012/2021/AMJ, 0001/2024/RDP, 0001/2024/AKP, 0092/2022/A2,0144/2022/A3)], and Shenzhen-Hong Kong-Macao Science and Technology Fund (Category CSGDX20220530111203020).

The original version of this article has been updated.

