# Supplementary material for: Improvement on mitochondrial energy metabolism of Codonopsis pilosula (Franch.) Nannf. polysaccharide
Source: Front Pharmacol. 2025 May 30;16:1545356. doi: 10.3389/fphar.2025.1545356 (PMC12162984; doi:10.3389/fphar.2025.1545356)
Supplement: Supplementary file 1 [file Supplementaryfile1.pdf]

Table 1

| Group | Dose (mg/kg/day) | AEC | ATP/ADP | ATP/AMP |
|---|---|---|---|---|
| Model | - | 0.67±0.05 | 1.18±0.14 | 3.22±1.12 |
| Normal | - | 0.75±0.07[b] | 1.39±0.21[a] | 10.46±2.68[c] |
| CPP | 200 | 0.72±0.04[a] | 1.28±0.14 | 6.28±2.76[b] |
| | 300 | 0.74±0.06[a] | 1.32±0.15[a] | 8.89±2.33[c] |
